# GABAergic Control of Critical Developmental Periods for Anxiety- and Depression-Related Behavior in Mice

**DOI:** 10.1371/journal.pone.0047441

**Published:** 2012-10-11

**Authors:** Qiuying Shen, Thomas Fuchs, Nadia Sahir, Bernhard Luscher

**Affiliations:** 1 Department of Biology, Pennsylvania State University, University Park, Pennsylvania, United States of America; 2 Department of Biochemistry & Molecular Biology, Pennsylvania State University, University Park, Pennsylvania, United States of America; 3 Department of Psychiatry, Pennsylvania State University, College of Medicine, Hershey, Pennsylvania, United States of America; 4 Center for Molecular Investigation of Neurological Disorders, Pennsylvania State University, University Park, Pennsylvania, United States of America; McLean Hospital/Harvard Medical School, United States of America

## Abstract

Vulnerability for anxiety and depressive disorders is thought to have origins in early life and is increasingly recognized to involve deficits in GABAergic neurotransmission. Mice that were rendered heterozygous for the γ2 subunit gene of GABA_A_ receptors (GABA_A_Rs) show behavioral, cognitive, neuroendocrine and pharmacologic features expected of a mouse model of melancholic anxious depression, including reduced survival of adult-born hippocampal neurons. Here we embarked on elucidating the developmental substrate underlying this phenotype, focusing on the Elevated Plus Maze and Forced Swim Test as relevant behavioral paradigms. In a first series of experiments using hemizygous tamoxifen-induced genetic inactivation of a floxed γ2 genomic locus we show that reducing the gene dosage at postnatal days (P)13/14 but not P27/28 results in altered behavior in both of these tests in adulthood, reminiscent of the anxious-depressive phenotype previously described for global heterozygous mice. However, in contrast to global heterozygous mice, the behavioral changes induced by γ2 subunit knockdown at P13/14 occurred without changes in adult hippocampal neurogenesis, indicating that altered neurogenesis is not an absolute prerequisite for anxiety- and depression-related behavior in this model. In a separate series of experiments using a pharmacological approach, acute but transient potentiation of GABA_A_Rs with diazepam uncovered distinct developmental vulnerabilities for altered behavior in the Elevated Plus Maze and Forced Swim Test, respectively. Specifically, diazepam given during P10-16 but not during later weeks resulted in increased anxiety-like behavior in adulthood, while diazepam administered during P29-35 but not earlier nor later resulted in increased immobility behavior in adulthood. We conclude that anxiety-like behavior in the Elevated Plus Maze and behavioral despair-like immobility in the Forced Swim Test are controlled by separate postnatal critical periods characterized by distinct developmental sensitivity to manipulation of GABAergic transmission via γ2 subunit-containing GABA_A_Rs.

## Introduction

Extensive comorbidity among major depressive disorder (MDD) and anxiety disorders suggests related disease etiologies [1], [2]. Reduced brain concentrations of the inhibitory neurotransmitter γ-aminobutyric acid (GABA) and altered function or reduced expression of its principal receptors (GABA_A_Rs) are increasingly implicated in both types of disorders [3]. GABA_A_Rs serve as the principal receptors mediating neural inhibition in the brain. Structurally they are heteropentameric chloride channels composed of α1-6, β1-3, γ1-3, δ, ε, π, θ, and ρ1-3 subunits [4]. The γ2 subunit-containing subtypes of GABA_A_Rs (containing two α1-3 or 5 subunits, two β1-3 subunits and one γ2 subunit) are of particular interest as they mediate the behavioral actions of benzodiazepines (BZs) [5], [6]. Mice that are heterozygous for the γ2 subunit gene (*gabrg2*, γ2^+/−^) exhibit anxious-depression-related behavior, including cognitive, cellular, neuroendocrine and pharmacological alterations expected of a mouse model of melancholic major depression [7], [8], [9]. Behavioral changes reminiscent of those of γ2^+/−^ mice have also been described for mice that lack the α2 subunit of GABA_A_Rs [10], [11]. Clinical and animal experiments together indicate that pre- and/or postsynaptic functional deficits in GABAergic transmission may be causative for anxiety and mood disorders (reviewed in [3], [12], [13]).

Conditional hemizygous inactivation of the γ2 gene in the embryonic telencephalon of mice results in an anxious-depressive-like phenotype in adulthood comparable to that of global γ2^+/−^ mice [8], [9]. By contrast, forebrain-specific heterozygous inactivation of the γ2 gene that is developmentally delayed to approximately the fifth postnatal week is without behavioral consequences [8]. These findings point to an embryonic or postnatal developmental origin of anxiety- and depression-related behavior. Consistent with these preclinical findings there is also significant clinical evidence that the vulnerability for anxiety and major depression is increased during early life [14]. Moreover, the two types of disorders have been proposed to have distinct developmental origins [15], [16].

Defects in adult hippocampal neurogenesis have been proposed as a possible cellular substrate for anxiety- and depression-related behavior [17], although several studies suggest that blocking neurogenesis is insufficient to affect performance in corresponding behavioral paradigms [18], [19], [20], [21] (for review see [22], [23], [24]). GABAergic transmission via γ2 subunit-containing GABA_A_Rs plays a key role in regulating cell fate decisions in adult quiescent stem cell niches [25] and in the dendritic maturation and synaptic integration of adult-born neurons [26], [27] (reviewed in [28], [29]). In particular, anxious-depression-related behavior of γ2 subunit-deficient mice with global or embryonically induced GABA_A_R deficits show a marked reduction in the survival of adult-born hippocampal neurons, whereas hippocampal neurogenesis is unaffected in behaviorally normal mice with a developmentally delayed GABA_A_R deficit [8]. These experiments suggested that the manifestation of anxiety- and depression-related behavior in GABA_A_R-deficient mice might involve deficits in adult hippocampal neurogenesis (reviewed in [3]).

Differences in the rate of activity-dependent maturation of neural circuits are thought to underlie brain function-specific critical periods, i.e. developmental periods during which a certain disturbance has a significantly greater impact than the same event later in life [30]. A gradual shift from GABAergic neural excitation of immature neurons in the developing brain to mainly inhibitory effects of GABA on mature neurons in adulthood is thought to underlie intrinsic activity-dependent control of neural circuit formation and thereby to provide a neurochemical basis for critical developmental periods of brain disorders [29], [31], [32]. As a case in point, the critical period underlying ocular dominance plasticity of the visual cortex is sensitive to both intra-cortical genetic reductions in GABA synthesis and pharmacological potentiation of GABA_A_R function with the BZ site agonist diazepam (DZP) [33]. Based on these data it was proposed that the potential for functional plasticity of neural circuits is retained until a certain level of circuit maturity, defined by a GABAergic inhibitory threshold, is attained [32], [34].

Here we present the results from two independent studies designed to define critical developmental periods during which perturbations of intrinsic GABAergic neural activity in mice result in lasting behavioral changes in the Elevated Plus Maze (EPMT) and Forced Swim Test (FST), respectively. In addition, we extend our studies addressing the role of hippocampal neurogenesis in regulating such behavior. First, we employed tamoxifen-inducible heterozygous knockout of the γ2 subunit gene at different time points of postnatal brain development, using mice carrying a single copy of a floxed γ2 subunit gene locus (fγ2/+, [8]) and the CAGGCre-ER™ locus [35] encoding a ubiquitously expressed tamoxifen-inducible Cre-estrogen receptor fusion protein. These experiments narrowed the developmental period underlying altered EPMT and FST behavior to between the beginning of the third and end of fifth postnatal week. In addition, and contrary to previous global or embryonic reductions of the γ2 gene dosage in immature neurons [8], these experiments showed that postnatal GABA_A_R deficit-induced anxiety- and depression-related behavioral changes can occur independently of reduced hippocampal neurogenesis. Second, we used pharmacological potentiation of GABA_A_Rs with DZP to transiently but more abruptly perturb brain development during distinct postnatal temporal windows predicted to underlie normal anxiety- and depression-related behavior in adulthood. Our experiments show that genetic impairment and pharmacologic potentiation of GABAergic transmission between the second and fifth postnatal week of mice have comparable lasting and detrimental consequences on anxiety- and depression-related behavior in adulthood. Moreover, the DZP treatment experiments identify two distinct developmental critical periods that selectively underlie behavior in the EPMT and FST, respectively.

## Materials and Methods

### Animals

All mice used for this study were backcrossed onto the 129X1/SvJ genetic background (>5 generations, previously named 129SvJ) and produced in our own breeding colony with food and water available ad libitum, on a 12 h:12 h light-dark cycle. GABA_A_R γ2 subunit global knockout (KO; γ2^−/−^ or γ2^+/−^) [6], [7] and floxed heterozygous γ2 (fγ2/+) mice [36] were generated in-house and genotyped as previously described [36]. CAGGCre-ER™ mice [also known as Tg(CAG-cre/Esr1*)5Amc/J] [35] were purchased from Jackson Laboratory (Bar Harbor, ME). ROSA26-YFP (R26Y) mice used as Cre reporters [37] were provided by Dr. A. J. Eisch (University of Texas Southwestern Medical Center). All mutant and control animals compared were produced as littermates. Accordingly, γ2^−/−^, γ2^+/−^ and wild-type (WT) mice used to analyze cortical neurogenesis were produced by mating of γ2^+/−^ mice with γ2^+/−^ or WT mice. CAGGCre-ER™ X fγ2/+, fγ2/+, CAGGCre-ER™ and WT mice were produced by mating hemizygous CAGGCre-ER™-transgenic mice with fγ2/+ mice. All mice were weaned between P20 and P22. The ages of mice given in postnatal days (P) refer to the exact age of all mice in a group at the time of treatment. The ages of mice indicated in weeks refer to the age of mice pooled from multiple litters in number of weeks ±3 days, at the time of testing. All animal experiments were performed in accordance with NIH guidelines and approved by the Institutional Animal Care and Use Committee (protocols # 29296, 32425) of the Pennsylvania State University.

### Tamoxifen Treatment and Quantitation of Tamoxifen-induced Recombination Efficacy

Cre-mediated recombination of floxed target genes was induced by a total of two injections of tamoxifen (TAM, LP Biomedicals LLC, Solon, OH), one day apart. The daily dosage was 180 mg/kg per day (i. p., 30 mg/ml) and the drug was emulsified in ethanol:sunflower seed oil (1∶9). As part of the drug treatment procedure the pups were temporarily transferred to a new cage (for maximally 5 min) until all mice of a litter had been treated. For P13/14 TAM and vehicle treatment this procedure included temporarily separating the pups for maximally 5 minutes from their mother.

### Analyses of Tamoxifen Induced Recombination Efficiency

CAGGCre-ER™ X R26Y mice were injected with tamoxifen at P13/14 or P27/28 as described above and anesthetized and trans-cardially perfused with 4% paraformaldehyde in phosphate buffered saline (PBS) at six weeks of age. The brains were postfixed for 16 h in the same fixative, rinsed in PBS three times and stored in PBS containing 0.05% sodium azide at 4°C until all brains were ready for sectioning. Coronal sections (50 µm) were cut with a Vibratome (Vibratome, St. Louis, MO) using a brain matrix (Electron microscopy sciences, Hatfiled, PA) for reproducible positioning of brains and processed for immunofluorescent staining using rabbit anti-green fluorescence protein [GFP (1∶1000), recognizing YFP, Invitrogen, Carlsbad, CA] antiserum and goat anti-doublecortin (DCX, 1∶250) antibody (Santa Cruz, Santa Cruz, CA), developed with anti-rabbit Alexa 488 and anti-goat Cy3 secondary antibodies (1∶500. Jackson ImmunoResearch), and rinsed in nuclear stain DRAQ5 (1∶1000, Cell Signaling Technology, Boston, MA) or DAPI (dihydrochloride salt in water, 14.3 mM, Invitrogen). The percentage of DRAQ5- or DCX-positive cells that colocalized with YFP immunofluorescence in regions of interest was determined by counting of cells in confocal images. Optical Z-plane sectioning (1 µm steps) was used to ensure that colocalized signals belonged to the same cells. The method used for analyses of TAM-induced recombination of the fγ2 locus by PCR is provided in Materials and Methods S1.

### Diazepam Treatment

DZP (Sigma Aldrich, St. Louis, MO) was administered p.o. as a suspension in 0.3% tween 80 in saline for a dosage of 1 mg/kg/day for treatment during P10-16 or 2 mg/kg/2 days for treatments during P14-28, P22-28, P29-35, and P50-56. Control mice were gavaged identically with vehicle alone. Drug and vehicle treated mice were briefly transferred to a new holding cage as was done for TAM treatment. The behavioral effects of DZP administered at 1 mg/kg/day from P10-16 were comparable to those of DZP administered at 2 mg/kg every other day during P14-28. Therefore, for practical reasons all other DZP treatments used a dose of 2 mg/kg every two days. Doses of 1–2 mg/kg DZP have acute anxiolytic but no sedative effects [7], [38]. Notably, higher sedative doses of DZP (5–10 mg/kg) were avoided as they affect locomotion of mice in adulthood [39].

### Behavioral Assessment

All experiments were performed with female mice, the gender that in humans is more vulnerable to anxiety and mood disorders [40], [41]. The mice were group housed 6–12 animals per large cage (56×40×20 cm) in a female only room with a 12 h:12 h light-dark cycle (light from midnight to noon), separated by genotype if applicable, starting in the fourth postnatal week. We found that these conditions suppress estrus cycling thereby alleviating concerns with estrus-dependent behavioral variation (unpublished observation). Behavioral testing of TAM-treated mice was done under red light between 8 and 11 weeks of age, one test per week, at least 72 h after the last cage change, and starting 1 h after onset of the dark phase of the light-dark cycle. Testing of DZP-treated mice was done under the same conditions between 8 and 15 weeks of age, no sooner than four weeks after the last DZP injection. Before testing, the test equipment was routinely exposed to same sex trial mice to saturate the maze with mouse odor. The test apparatus was wiped clean of excrement after each mouse. Testing routinely started with an Open Field Test (OFT), followed by EPMT [42] and a FST [43] with the experimenter blinded to experimental conditions. The FST of P29-35 DZP-treated mice was followed by a Tail Suspension Test (TST) [44]. No OFTs were done for P28/29 TAM-treated and P14-28 and P22-28 DZP-treated mice.

The OFT was used to assess possible alterations in baseline locomotion that might affect EPMT and FST measures. The mice were placed into the corner of a novel open field arena (50×50 cm), and the total distance travelled over 15 min was recorded with an Ethovision system (Noldus Information Technology, Inc., Leesburg, VA). Given that the OFT was performed under red light it cannot be used to assess neophobia. The EPMT [7] was used to assess anxiety-like aversion to explore a novel elevated open space as opposed to an enclosed space. The test arena consisted of an elevated crossbar (30 cm length per arm X 5 cm wide X 40 cm tall) with two walled (20 cm, clear Plexiglas) and two open arms. The edges of the open arms were surrounded by a 4 mm wide and 2 mm high trim to prevent mice from falling off the maze. The mice were placed onto the center square of the maze and videotaped for 5 min. The % closed arm entries, the % time spent on open arms (% open arm duration) and the number of closed arm entries were recorded. The FST [45] was used to assess behavioral immobility of mice in response to exposure to an inescapable stressful situation. Reduced immobility in this test has predictive validity for antidepressant drug action [46]. Conversely, increased immobility in this test has been proposed as an index of behavioral despair [46] (however, see Discussion) and is correlated with depressive-like behavioral, endocrine and pharmacological characteristics in γ2^+/−^ mice [9]. The mice were lowered into a plastic beaker 19 cm in diameter and 27 cm deep and filled to a height of 18 cm with 24–26°C water and video recorded for 6 min. We recorded the real time spent swimming until the first floating episode and the cumulative time spent immobile during the final 4 min, using a 5 s interval sampling method. The TST [44], [47] was used to confirm FST results of P29-35 DZP-treated mice. It assesses escape behavior in an inescapable stressful situation and has predictive validity for antidepressant drug action similar to the FST. The mice were suspended by their tails from a stainless steel rod (affixed with adhesive tape) that was positioned 30 cm above the floor of an apparatus (50×50×45 cm) consisting of two 25 cm wide compartments separated by an opaque PVC board. Two mice were videotaped side-by-side and the latency to assume an immobile posture and the total immobility time during the entire 6 min trial were recorded.

### Analyses of the Survival and Differentiation of Adult-born Hippocampal Neurons

Female P13/14 TAM-treated CAGGCre-ER™ X fγ2/+ mice and littermate controls were injected with the DNA synthesis marker 5-bromo-2′deoxyuridine (BrdU, 3×75 mg/kg, administered i.p. in 2 h intervals as a solution of 10 mg/ml BrdU in 0.9% NaCl) at P63 and transcardially perfused at P91 with 4% paraformaldehyde in 0.1 M phosphate buffered saline (PBS, pH 7.4) postfixed for 12 h in the same solution, and equilibrated in 30% sucrose for at least one day. Free-floating sections cut coronally (50 µm) by Vibratome (Vibratome, St. Louis, MO) were permeabilized with 1% Triton X-100 in PBS, incubated in 2N HCl for 30 min at 37°C, and washed for 5 min in 0.1 M sodium borate (Na_2_B_4_O_7_) at room temperature followed by four times 5 min in PBS. They were stained with a rat antiserum against BrdU (1∶500; Accurate Chemical, Westbury, NY) and a monoclonal antibody for neuronal-specific nuclear protein (NeuN)(1∶1000; Chemicon, Temecula, CA), followed by appropriate Cy3- and Alexa 488-coupled secondary antibodies (1∶500, Jackson ImmunoResearch, West Grove, PA). The number of BrdU and BrdU/NeuN positive neurons in the subgranule cell layer of confocal images across sections of the entire bilateral hippocampus were counted as described [8]. Optical sectioning was used to verify colocalization of BrdU and NeuN. The methods for analyses of production, migration and survival of embryo-derived neocortical and hippocampal neurons are provided in Materials and Methods S1.

### Statistical Analyses

Statistical analyses were conducted using Minitab15 (Minitab Inc., State College, PA). Simple comparisons of two group means of behavioral tests were done by two-sample two-tailed t-tests. The latency to immobility data of FSTs and TSTs were all log transformed to satisfy the homogeneous variance assumption. One-way analyses of variance (ANOVAs) were used for comparison of behavioral data of multiple genotypes, followed by *post hoc* analyses of group means by Dunnett’s tests. In the case of large experimental groups of mice that had to be split into two subgroups for testing over two days, the two subgroups were treated as blocks with the blocking effect treated as a random factor. Group means of cell counts of morphological analyses were compared by Mann-Whitney tests (for two groups) or Kruskal-Wallis tests (three groups). The latter were followed up by pairwise comparisons using Mann-Whitney tests.

## Results

### Unaltered Neocortical Development of γ2^+/−^ Mice

Previous analyses of mice with developmentally distinct conditional hemizygous deletions of the γ2 subunit locus indicated that the anxious-depressive-like phenotype of these mice had a developmental origin between embryonic day 10 (E10, corresponding to the start of cortical neurogenesis [48]) and approximately the fifth postnatal week in the forebrain [8]. However, the densities of E12.5 and E15.5-derived neocortical neurons that had migrated to the subplate and layer II/III of the neocortex, respectively of γ2^+/−^ and γ2^−/−^ mice were indistinguishable from values of WT littermates (Figure S1a–c). Similarly, the densities of E15.5-derived neurons surviving to P21 in the CA1 and CA3 region and dentate gyrus of the hippocampal formation were unaltered in γ2^+/−^ vs. WT littermates (Figure S1d). The data suggested that previously described behavioral alterations of γ2^+/−^ mice had most likely a postnatal developmental origin.

### Characterization of TAM-mediated Recombination Patterns

Given the evidence that embryonic corticogenesis was unaffected even in γ2^−/−^ mice we from here onwards focused on delineating critical periods of γ2^+/−^ mice during postnatal development. We employed a TAM-inducible global KO strategy, making use of mice that are heterozygous for a *gabrg2* locus containing lox P sites (fγ2/+) [36] and a CAGGCre-ER™ transgenic line that expresses a TAM-inducible Cre recombinase (Cre-ER™) [35]. Comparing the behavioral consequences of Cre-mediated recombination induced by TAM at different developmental time points requires that the recombination efficiency is independent of the age at which recombination is induced. We used Cre-mediated recombination of the R26Y locus as a proxy for recombination of the fγ2 locus. Double transgenic offspring of CAGGCre-ER™ X R26Y matings were injected with TAM on two sequential days (P13/14 or P27/28, respectively) and the brains analyzed at six weeks of age for recombination in the dentate gyrus, CA1 region of the hippocampus and frontal cortex, i. e. primary brain regions implicated in anxious and depression-related behavior of global γ2^+/−^ mice (Discussion). Immunostaining of the dentate gyrus granule cell layer revealed similar recombination efficiency at P13/14 and P27/28, independent of the time point of TAM injection (YFP-positive cells as a fraction of total number of cells visualized with DRAQ5 or DAPI nuclear stains: P13/14, 33.1±10.6%; P27/28, 35.8±4.4%, p>0.05, n = 4–6 mice, Figure S2). The average recombination efficiency of P13/14 and P27/28 TAM-treated mice was homogenous across brain regions as indicated by similar recombination efficiencies in the hippocampal CA1 region (38.4±2.6%) and frontal cortex (31.5±8.6%, p>0.05, n = 3–6 mice, Mann-Whitney tests). Furthermore, analyses of genomic DNA of TAM-treated CAGGCre-ER™ X fγ2/+ mice by PCR indicated that conditions identical to those used to induce recombination of the R26Y locus of CAGGCre-ER™ X R26Y mice reliably resulted in recombination of the fγ2 locus within 24–48 h of TAM treatment (Figure S3).

### Behavioral Characterization of TAM-treated Control Mice

**Figure 1:**
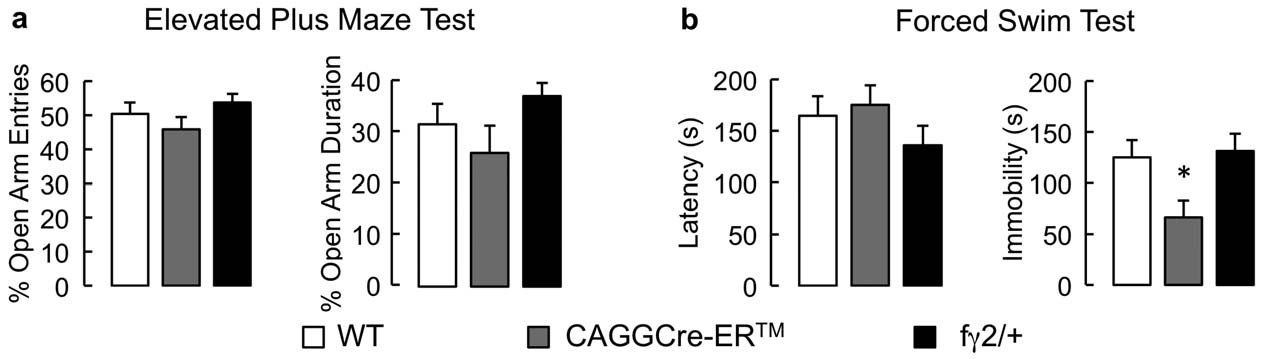
Behavioral effects of tamoxifen in the EPMT and FST independent of target gene recombination. TAM was injected into CAGGCre-ER™, fγ2/+ and WT littermates at P13/14 or P27/28 to test for TAM- and Cre-induced behavioral effects at 8–10 weeks of age that occurred independently of recombination of the fγ2 locus. **a)** In the EPMT, the CAGGCre-ER™ and fγ2/+ mice visited open arms and stayed in the open arms as much as WT mice. **b)** In the FST, the behavior of fγ2/+ mice was indistinguishable from WT mice. CAGGCre-ER™ mice showed a normal latency to immobility and reduced immobility duration compared to TAM-treated fγ2/+ and WT mice. All values represent group means ± SEM. *p<0.05, Dunnett’s test.

**Figure 2:**
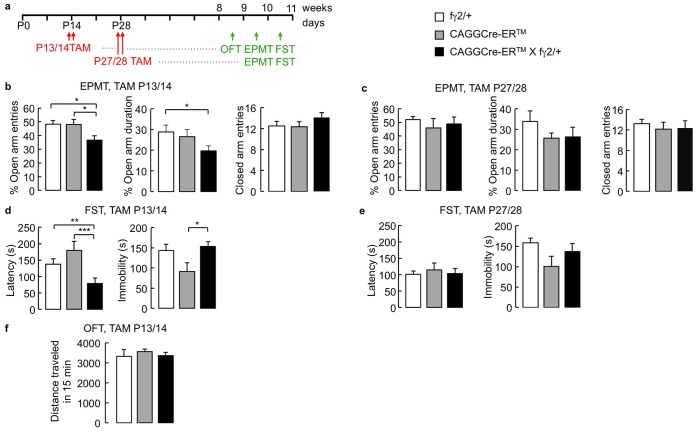
Developmental control of anxious depressive behavior induced by tamoxifen-induced Cre-mediated recombination of the fγ2 locus. **a)** Experimental time line. Littermates representing all three genotypes were injected with TAM at P13/14 or P27/28, followed by behavioral testing starting after 8 weeks of age. **b)** In the EPMT, CAGGCre-ER™ X fγ2/+ mice treated with TAM at P13/14 visited the open arm less often than identically treated fγ2/+ and CAGGCre-ER™ controls. Moreover, P13/14 TAM-treated CAGGCre-ER™ X fγ2/+ mice spent less time on the open arms than identically treated fγ2/+ controls, while the number of closed arm entries was unaffected. **c)** CAGGCre-ER™ X fγ2/+ mice treated with TAM at P27/P28 were indistinguishable from identically treated fγ2/+ and CAGGCre-ER™ littermate controls with respect to % open arm entries, % time spent on open arms, and number of closed arm entries. **d)** In the FST, the latency of P13/14 TAM-treated CAGGCre-ER™ X fγ2/+ mice to float was reduced compared to identically treated CAGGCre-ER™ and fγ2/+ littermate controls. Moreover, P13/14 TAM-treated CAGGCre-ER™ X fγ2/+ mice showed increased immobility compared to identically treated CAGGCre-ER™ controls. **e)** P27/28 TAM-treated CAGGCre-ER™ X fγ2/+ mice were indistinguishable from identically treated fγ2/+ and CAGGCre-ER™ littermates with respect to both latency to immobility and immobility duration. **f)** In the OFT, the distance traveled of P13/14 TAM-treated CAGGCre-ER™ X fγ2/+ mice over a period of 15 min was indistinguishable from that of identically treated CAGGCre-ER™ and fγ2/+ littermates. All values represent group means ± SEM. *p<0.05, **p<0.01, ***p<0.001, Dunnett’s tests.

We showed previously that anxiety- and emotion-related behavior of pseudo-WT fγ2/+ mice was indistinguishable from WT controls [8], in agreement with normal expression of γ2-containing GABA_A_Rs in fγ2/fγ2 mice [36]. Recombination of floxed target genes of CAGGCre-ER™-transgenic mice relies on TAM-induced translocation of Cre protein to the nucleus. High levels of nuclear Cre activity can lead to unspecific recombination of genomic DNA at cryptic loxP sites with Cre-dependent long-term toxic consequences [49]. To address whether TAM-induced unspecific Cre-activity or TAM alone might affect baseline behavior of TAM-treated CAGGCre-ER™ mice, TAM was injected into CAGGCre-ER™, fγ2/+ and WT littermates at either P13/14 or P27/28 followed by behavioral testing between 8 and 10 weeks of age. P13/14- and P27/28-TAM-treated mice were indistinguishable in any of the test parameters; therefore the data for the two treatment ages were combined. TAM-treated CAGGCre-ER™, fγ2/+ and WT mice analyzed in the EPMT (Figure 1a) and FST (Figure 1b) were indistinguishable from each other with respect to % open arm entries [F(2,31) = 0.2, p = 0.82] and % open arm duration [F(2,31) = 0.37, p = 0.70, n = 7–14] in the EPMT, as well as latency to immobility in the FST [F(2,43) = 2.2, p = 0.12, n = 14–16, ANOVAs). However, there was a significant genotype effect on FST immobility duration [F(2, 43) = 4.32, p = 0.02]. Posthoc analyses confirmed that the immobility duration of TAM-treated CAGGCre-ER™ mice was reduced compared to that of identically treated fγ2/+ [q (43, 3) = 2.715, p<0.05, n = 14–16] and WT mice [q (43, 3) = 2.49, p<0.05, n = 14–16, Dunnett’s test] (Figure 1b). This Cre-transgene-mediated effect was independent of a floxed locus and therefore likely due to unspecific Cre-mediated recombination of cryptic loxP sites [50]. Importantly, the direction of this change in immobility duration observed in TAM-treated CAGGCre-ER™ vs. TAM-treated fγ2/+ mice was the inverse of the change described below for P13/14 TAM-treated CAGGCre-ER™ X fγ2/+ vs. CAGGCre-ER™ mice (Figure 2), which is consistent with an unspecific mechanism entirely different from GABA_A_R deficit-induced increases in immobility.

### Heterozygosity of the γ2 Subunit Gene Induced at P13/14 but not P27/28 Results in Heightened Anxiety- and Depression-related Behavior in Adulthood

We first analyzed the consequences of γ2 subunit gene deletion induced at P13/14 or P27/P28 on anxiety-related behavior in the EPMT in adulthood. CAGGCre-ER™ X fγ2/+ mice and CAGGCre-ER™ and fγ2/+ littermate controls were treated with TAM either at P13/14 or P27/P28 and subjected to behavioral testing between 8 and 10 weeks of age (Figure 2a). In the EPMT, P13/14 TAM-treated mice showed a significant genotype effect for the parameter % open arm entries [F(2,67) = 4.03, p = 0.022, ANOVA] and a corresponding strong trend for % open arm duration [F(2,27) = 2.82, p = 0.077, ANOVA]. Posthoc analyses confirmed that CAGGCre-ER™ X fγ2/+ mice visited the open arms less often than fγ2/+ and CAGGCre-ER™ littermates and that they spent less time on the open arms than fγ2/+ controls (% open arm entries, CAGGCre-ER™ X fγ2/+ vs. fγ2/+, q (67, 3) = 2.64, p<0.05, CAGGCre-ER™ X fγ2/+ vs. CAGGCre-ER™, q (67, 3) = 2.37, p<0.05, n = 19–32; % open arm duration, CAGGCre-ER™ X fγ2/+ vs. fγ2/+, q (27, 3) = 2.33, p = 0.05, n = 9–13, Dunnett’s tests) (Figure 2b). By contrast, P27/28 TAM-treated CAGGCre-ER™ X fγ2/+ mice tested analogously were not different from fγ2/+ and CAGGCre-ER™ littermate controls [% open arm entries, F(2, 41) = 0.61, p = 0.548; % open arm duration, F(2, 41) = 1.64, p = 0.206, ANOVAs] (Figure 2c). The data indicate that P13/14 bot not P27/28 TAM treatment of CAGGCre-ER™ X fγ2/+ mice results in anxiety-like behavior in adulthood.

Next we compared TAM-treated CAGGCre-ER™, fγ2/+ and CAGGCre-ER™ X fγ2/+ mice in the FST. Reduced immobility in this test is predictive of antidepressant drug activity [43], [45], while increased immobility in γ2^+/−^ mice is associated with anhedonia-like reductions in sucrose consumption, hypercortisolism and increased behavioral sensitivity to diazepam, fluoxetine and desipramine indicative of an anxious-depressive state [7], [9]. P13/14 TAM-treated mice showed significant genotype effects for latency to immobility [F(2,46) = 10.47, p<0.001] and immobility duration [F(2,46) = 3.68, p<0.05, ANOVAs]. Posthoc tests confirmed that CAGGCre-ER™ X fγ2/+ mice showed a reduced latency to immobility compared to fγ2/+ and CAGGCre-ER™ controls [CAGGCre-ER™ X fγ2/+ vs. fγ2/+, q (46, 3) = 3.80, p<0.01; CAGGCre-ER™ X fγ2/+ vs. CAGGCre-ER™, q (46, 3) = 4.23, p<0.001, n = 14–20, Dunnett’s tests] (Figure 2e). The immobility duration of P13/14 TAM-treated CAGGCre-ER™ X fγ2/+ mice was also increased but only when compared to CAGGCre-ER™ mice [q (46, 3) = −2.55, p<0.05, n = 14–16] and not when compared to fγ2/+ controls (p>0.05, Dunnett’s tests). Importantly, the aforementioned lox P site-independent effect of TAM treatment on immobility duration of CAGGCre-ER™ mice (Figure 1b) indicates that CAGGCre-ER™ but not fγ2/+ mice serve as the appropriate control for the parameter immobility duration. Similar to the EPMT, the behavior of P27/28 TAM-treated CAGGCre-ER™ X fγ2/+ mice in the FST was indistinguishable from that of identically treated fγ2/+ and CAGGCre-ER™ littermate controls [latency, F(2,42) = 0.38, p = 0.689; immobility, F(2,42) = 2.37, p = 0.106, ANOVAs] (Figure 2e). Altered behavior of P13/14 TAM-treated CAGGCre-ER™ X fγ2/+ mice in the EPMT and FST was not due to a change in locomotion as indicated by the absence of genotype effects on closed arm entries in the EPMT [F(2,31) = 0.22, p = 0.8, ANOVA] (Figure 2a) and distance traveled during a 15 min OFT [F(2,25) = 0.44, p = 0.65, ANOVA] (Figure 2f). Thus, TAM-induced inactivation of the fγ2 gene of CAGGCre-ER™ X fγ2/+ mice at P13/14 but not P27/28 resulted in anxiety- and depression-related behavioral changes in the EPMT and FST similar to those previously described for mice with germ line or embryonically-induced GABA_A_R deficits [8], [9].

### Altered Behavior of P13/14 TAM-treated CAGGCre-ER™ X fγ2/+ Mice does not Involve Deficits in the Survival of Adult-born Hippocampal Neurons

**Figure 3:**
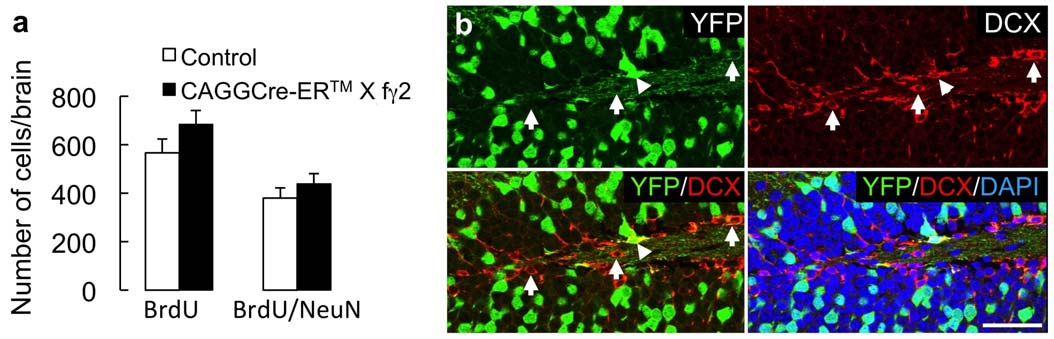
Quantitation of hippocampal neurogenesis and analyses of recombination in neural progenitors. **a)** P13/14 TAM-treated CAGGCre-ER™ X fγ2/+ mice and littermate controls (CAGGCre-ER™ and fγ2/+) were injected with BrdU at P63 and transcardially perfused at P91. The number of BrdU-positive or BrdU/NeuN doubly positive hippocampal granule cells quantitated in serial sections was indistinguishable in TAM treated CAGGCre-ER™ X fγ2/+ mice compared to identically treated littermate controls (mixed CAGGCre-ER™ and fγ2/+ genotypes). **b)** Cre-mediated recombination in adult-born hippocampal neurons of P13/14 TAM-treated CAGGCre-ER™ X ROSA26-YFP mice was analyzed at 6 weeks of age. The micrographs show a representative section immunostained for YFP (upper left panel, green), DCX (upper right, red), with merged images showing YFP/DCX-doubly positive cells (lower left) and the outlines of the granule cell layer stained with the nuclear stain, DAPI (lower right). Arrows point to DCX positive immature neurons lacking YFP that therefore were not subject to Cre-mediated recombination. The arrowhead points to a rare DCX/YFP positive immature neuron that was subject to Cre-mediated recombination. Scale bar, 50 µm.

We previously showed that the manifestation of heightened aversion to open arms in the EPMT and increased immobility in the FST of three different γ2-subunit-deficient mouse lines (γ2^+/−^, Emx1Cre X fγ2/+, CaMKIICre2834 X fγ2/+) was correlated with reduced survival of adult-born hippocampal neurons [8]. Here we assessed whether the same correlation between behavior and adult hippocampal neurogenesis could be extended to mice with GABA_A_R deficits induced at P13/14. CAGGCre-ER™ X fγ2/+ mice and CAGGCre-ER™ and fγ2/+ littermate controls were treated with TAM at P13/14, followed by metabolic labeling of replicating neural progenitor cells with BrdU at P63 and immunofluorescence analyses of brains at P91, allowing for four weeks of maturation of BrdU-labeled neurons. Interestingly, the numbers of BrdU-positive cells and BrdU positive hippocampal granule cells co-labeled with the mature neural marker NeuN in P13/14 TAM-treated CAGGCre-ER™ X fγ2/+ mice were indistinguishable from corresponding cell counts in identically treated CAGGCre-ER™ and fγ2/+ controls, indicating unaltered production and survival of adult generated granule cells (BrdU-positive cells, p = 0.53; BrdU/NeuN-doubly positive cells, p = 0.83, n = 5, Mann-Whitney) (Figure 3a). Consistent with these findings, immunostaining of the dentate gyrus of P13/14 or P27/28 TAM-treated CAGGCre-ER™ X R26Y mice at six weeks of age for the Cre reporter YFP and the immature neuronal marker doublecortin (DCX) revealed negligible rates of Cre-mediated recombination in adult-born neurons expressing DCX (% DCX-positive neurons colocalized with YFP averaged across both treatment groups: 5.03±1.2%, n = 6) (Figure 3b). Thus, heightened anxiety- and depression-related behavior of P13/14 TAM-treated CAGGCre-ER™ X fγ2/+ mice did not involve deficits in the production or survival of adult-born hippocampal neurons.

### Diazepam Treatment from P10 to P16 has Anxiogenic Effects on WT Mice Analyzed in Adulthood

**Figure 4:**
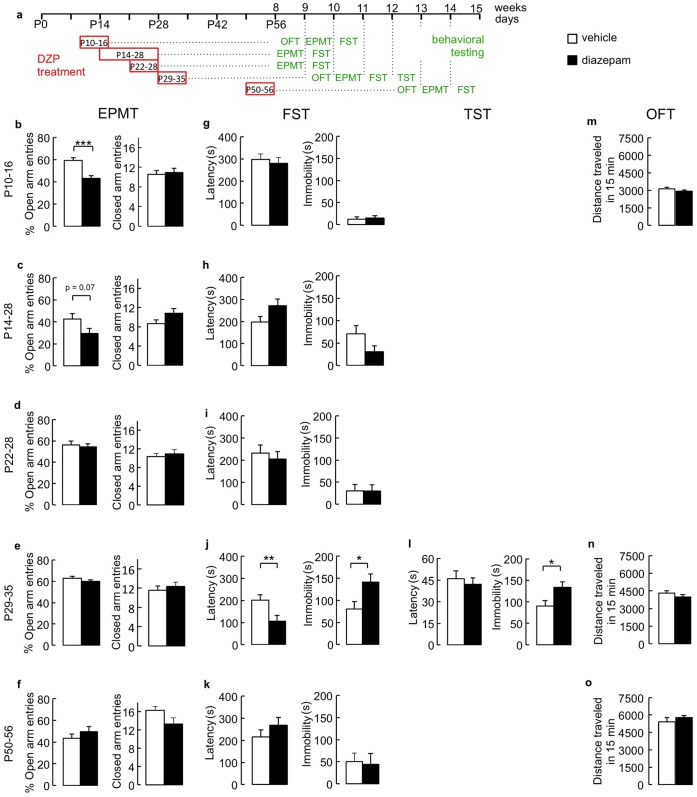
Behavioral consequences of diazepam treatment during one- or two-week postnatal developmental temporal windows. **a)** Schematic showing the schedule of DZP treatment and behavioral testing of the five cohorts of mice analyzed. DZP was administered during P10-16, P14-28, P22-28, P29-35 or P50-56, followed by behavioral testing between 8 and 11 weeks of age or, in the case of P29-35 and P50-56, between 4 and 9 weeks after the end of DZP treatment. **b–f)** In the EPMT, the percentage of open arm entries of P10-16 DZP- vs. vehicle-treated mice was reduced (b). Similarly, the percentage of open arm entries trended lower in P14-28 DZP-treated vs. vehicle treated mice (c). However, the number of closed arm entries was unaffected for both treatment periods, consistent with unaltered locomotion. By contrast, DZP treatment during P22-28, P29-35 and P50-56 was without long-term behavioral consequences in this test (d-f). **g–k)** When tested in the FST, the mice treated with DZP during P10-16, P14-28, P22-28 and P50-56 showed normal behavior compared to vehicle treated controls (g-i, k). By contrast, mice treated with DZP during P29-35 showed a reduced latency to immobility and increased immobility duration (j). **l)** Increased immobility of P29-35 DZP-treated mice observed in the FST was confirmed by increased immobility duration in the TST, although the latency to assume an immobile position was unchanged. **m–o)** The distance traveled of P10-16, P29-35 and P50-56 DZP-treated mice in a 15 min OFT was unaltered compared to controls, showing that locomotion was unaffected. *p<0.05, **p<0.01, ***p<0.001, t-tests.

Assuming that gradual loss of the γ2 protein following TAM injection occurred with a four to five day delay, we reasoned that reduced function of GABA_A_Rs between the end of the second and fifth postnatal week interferes with maturation of neural circuits underlying normal anxiety- and emotion-related behavior in adulthood. However, more precise mapping of critical periods by genetic means was hampered by the ill-defined temporal delay in loss of receptor function following TAM-induced gene deletion. Therefore, in a second study we tested whether the aforementioned results from genetic analyses could be corroborated by a pharmacological approach that is predicted to more abruptly perturb intrinsic neural activity-dependent developmental processes. Analysis of cortical ocular dominance plasticity indicates that critical developmental periods are not only sensitive to genetic reductions in GABA input but also to pharmacological potentiation of GABA_A_Rs with low, anxiolytic concentrations of DZP. Therefore, to test whether DZP treatment could be used to delimit critical periods for behavior in the EPMT and FST, WT mice were subjected to one- or two-week treatment with DZP or vehicle starting at different postnatal ages (P10-16, P14-28, P22-28, P29-35, P50-56), followed by behavioral analyses starting at eight weeks of age at least four weeks after the end of DZP treatment, one test per week (Figure 4a). Treatment with DZP during P10-16 resulted in anxiety-like behavior in the EPMT as indicated by the reduced % open arm entries compared to vehicle-treated controls [t(22) = 4.25, p<0.001, t-test] (Figure 4b). A strong trend in the same direction [t(19) = 1.92, p = 0.07, t-test] was also evident for P14-28 DZP-treated mice, representing a slightly delayed but temporally overlapping treatment window (Figure 4c). By contrast, DZP treatment during P22-28 (n = 8–10), P29-35 (n = 21–22) or P50-56 (n = 8–9) had no effect on behavior in the EPMT (p>0.05 for all three treatment periods, t-tests)(Figure 4d–f). The anxiogenic effects seen in P10-16 and P14-28 DZP-treated mice were not due to alterations in locomotion as indicated by unaltered closed arm entries in the EPMT [DZP(P10-16): p>0.05, n = 11,13; DZP(P14-28): p>0.05, n = 10,12, t-tests] (Figure 4b, c) and unaltered distance traveled of P10-16 DZP-treated mice in a 15 min. OFT (p>0.05, t-test) (Figure 4m). The data indicate that DZP treatment selectively during P10-16 had anxiogenic consequences in adulthood.

### Diazepam Administration from P29 to P35 Increases Immobility Responses in the FST in Adulthood

We next asked whether the critical period regulating anxiety-like behavior in the EPMT was the same or could be separated from that regulating immobility in the FST. Interestingly, DZP treatment selectively during P29-35 but not P10-16, P14-28, P22-28 or P55-56 resulted in a decreased latency to immobility, as well as increased immobility duration in this test [P29-35: latency to immobility, t(20) = 3.49, p<0.01, immobility duration, t(20) = 2.63, p<0.05; P10-16, 14-28, 22-28 and 55-56: n = 8–13, p>0.05 for all comparisons, t-tests] (Figure 4g–k). Increased immobility of P29-35 DZP-treated WT mice was also evident based on the increased immobility duration in the TST [t(20) = 2.1, p = 0.05, t-test], although the latency to immobility in this test was unaffected (p>0.05) (Figure 4l). Altered behavior in the FST and TST of P29-35 DZP-treated mice was associated with normal baseline locomotion, as indicated by the unaltered number of closed arm entries in the EPMT (Figure 4e) and the unaltered distance traveled in a 15 min OFT (Figure 4n) (p>0.05, n = 10, for both comparisons, t-tests). Locomotion was also unaffected by DZP treatment during P22-28 and P50-56 as evidenced by the unaltered number of closed arm entries in the EPMT for P22-28 and P50-56 DZP-treated mice (Figure 4d, f) and the normal distance traveled in the OFT of P50-56 DZP-treated mice (Figure 4o) (p>0.05 for all three comparisons, n = 8–10, t-tests). Collectively, the data indicate that anxiety-related behavior in the EPMT and immobility behavior in the FST are regulated independently by developmentally distinct critical periods that are similarly sensitive to treatment with diazepam.

## Discussion

We have presented the combined results of two independent studies assessing postnatal developmental periods during which perturbations of GABAergic transmission via γ2-containing GABA_A_Rs affect anxiety- and emotion-related behavior in adulthood.

In the first study, irreversible TAM-induced knockdown of the GABA_A_R γ2 subunit gene dosage identified a postnatal two-week period during which induction of a permanent GABAergic deficit led to increased anxiety- and depression-related behavioral measures in adulthood. Conditional TAM-induced recombination of the γ2 gene at P13/14 resulted in heightened anxious behavior in the EPMT and FST, similar to behavior previously reported for mice with global or embryonically reduced GABA_A_R expression [8]. By contrast, delayed TAM-induced inactivation of the γ2 locus at P27/28 was without significant behavioral consequences, similar to CaMKIICre2834-mediated delayed knockdown of the γ2 gene previously reported [8]. Under the conditions used for TAM-induced knockdown of the γ2 gene dosage, Cre-mediated recombination of the R26Y reporter was observed in about 35% of cells (Figure S2). Moreover, TAM-induced, CAGGCre-ER™-mediated recombination of the fγ2 locus was complete after less than 48 hours after the second injection of TAM (Figure S3). However, our attempts to quantitate the reduction of γ2 protein expression following TAM-induced recombination were not successful. Based on a recombination efficiency of one of two alleles in 35% of cells one might expect a reduction of γ2-containing GABA_A_Rs by 17%. However, in global γ2^+/−^ mice γ2-containing GABA_A_Rs were reduced on average across brain regions by approximately 25% only, i. e. about half of what was expected [7]. Accordingly, we estimate that TAM-treated CAGGCre-ER™ X fγ2/+ mice suffered from loss of the γ2 subunit in about 8.5% of GABA_A_Rs. Due to the known delay of TAM-induced and Cre-mediated recombination of floxed target genes (1–2 days) [35] (Figure S3) and an estimated extra 2–3 days needed for maximal decay of the γ2 subunit mRNA and protein, we estimate that the minimal vulnerability window during which reduced GABA_A_R gene dosage affects adult behavior maps to in-between P14 and P35 (the beginning of the 3^rd^ to the end of the 5^th^ postnatal week) of postnatal mouse development. Additional studies are needed to assess whether the phenotype of P13/14 TAM-treated CAGGCre-ER™ X fγ2/+ mice includes depression-related hypercortisolism, pharmacological alterations and anhedonia-like deficits previously reported for global γ2^+/−^ mice [9].

Our previous studies pointed to the hippocampus and cortex as likely substrates for GABA_A_R deficit-induced anxious-depression-related brain states. First, γ2^+/−^ mice with a global reduction of the γ2 gene dosage revealed functional deficits in postsynaptic GABA_A_Rs mainly in hippocampus and cingulate, piriform and frontal cortex (−25 to −35%), while such deficits were below threshold for reliable quantitation in the amygdala (<13%) [7], [8]. Second, anxious-depressive-like behavior of these mice was associated with impaired performance in trace fear conditioning and ambiguous cue fear conditioning [7], which are critically dependent on hippocampal structures [51], [52], while hippocampus-independent but amygdala-dependent delay conditioning was unaffected [7]. Third, a similar anxiety- and depression-related phenotype was also observed following selective reduction of γ2 subunit expression selectively in glutamatergic neurons of the telencephalon [8]. Fourth, the manifestation of this behavioral phenotype in different conditional or global γ2-deficient strains of mice was associated with deficits in the long-term survival of adult-born hippocampal granule cell neurons [8], which encouraged speculation that deficits in hippocampal neurogenesis might contribute to anxious depressive behavior of γ2^+/−^ mice. Here, we showed that selective knockdown of γ2 expression in two-week-old mice results in a comparable anxiety- and depression-related phenotype that did not involve altered production or survival of hippocampal granule cells. By extension, the aforementioned deficits in hippocampal neurogenesis of global γ2-deficient mice [8] are likely to represent an epiphenomenon of GABAergic deficit-induced anxiety- and depression-related behavior. These results complement and extend a number of other studies that concluded that reduced hippocampal neurogenesis was insufficient to induce anxious or depressive-like behavior [18], [19], [20], [21]. However, there is also evidence that blocking hippocampal neurogenesis may be sufficient to induce heightened anxiety- [53], [54] and depression-related behavior [17].

In a second study, we used potentiation of GABA_A_Rs with low concentrations of DZP to more precisely map critical developmental periods involved in establishing anxiety- and depression-related behavior. These experiments identified P10-16 and P29-35 as two critical periods that separately and specifically control anxiety-related (EPMT) and immobility (FST) behavior, respectively in adulthood. The idea that critical periods may be sensitive to both a reduction and potentiation of GABAergic transmission refutes the intuitive presumption that opposite manipulations must have opposite outcomes. Previous studies of ocular dominance plasticity of the visual cortex suggested that both genetic reduction of GABA synthesis and pharmacological potentiation of GABA_A_Rs with diazepam perturb sensory input-mediated ocular dominance plasticity [32]. Similarly, we here provide evidence that the developmental substrate for anxiety and depression-related behavior is sensitive to both genetic impairment and pharmacological potentiation of GABAergic transmission through GABA_A_Rs. Both types of manipulations relied on altering the function of γ2-containing GABA_A_Rs and thereby likely targeted similar neuroanatomical substrates. We postulate that both genetic impairment and pharmacological potentiation of GABA_A_Rs interfered with neural activity-dependent processes that normally drive the development of neural circuits underlying anxiety and depression-related behavior.

Our study is subject to several limitations. First, it relied principally on the EPMT and FST as proxies of anxiety- and depression-related behavior. The EPMT has face and construct validity for generalized anxiety, as well as high predictive validity for anxiolytic drug action [55]. By contrast, the validity of the FST (and the closely related TST) for depressive disorders is largely derived from its predictive validity for antidepressant drug action [55], [56], although the test has also been proposed as a measure of depression-related behavioral despair [57]. Therefore, future experiments will need to address whether the critical periods identified for FST immobility extend to other behavioral measures with face and construct validity for specific aspects of depression such as reduced reward sensitivity or increased stress sensitivity [58]. A second limitation is due to the fact that mice treated with TAM or DZP at different ages were part of independent cohorts that were treated and tested independently. Therefore, behavioral alterations attributed to different TAM or DZP treatment ages could be corroborated by differences in baseline behavior due to slight variation in experimental conditions. Future verification will require comparison of groups of animals produced as a single large cohort that were subjected to treatment at different ages and behaviorally tested on the same day. Third, we cannot exclude that drug withdrawal-induced stress and stress-induced increases rather than DZP-induced decreases in neural excitability contributed to the long-term behavioral consequences of DZP treatment. Lastly, the behavioral effects of gene knockout or DZP treatment might be corroborated by interactions with handling-induced stress [59].

The incomplete correspondence of sensitive periods mapped by genetic and pharmacologic methods might in part reflect the fact that the first approach relied on gradually and modestly reducing GABAergic input, thereby allowing for compensatory adaptations of neural excitability, while DZP treatment affects GABAergic input instantaneously and probably more potently. The slower time course and reduced potency of the genetic vs. pharmacologic manipulation may also explain the unaltered behavior of TAM28/29-treated CAGGCre-ER™ X fγ2/+ mice, which based on results of the DZP-treatment experiment would be predicted to show increased immobility in the FST.

The anxiety–related critical period identified here (P10-16) maps to within a larger temporal window (P5-21) previously implicated in the developmental programing of anxiety by analyses of 5-HT1A receptor knockout mice [60]. Similar early programming of anxiety-related behavior is also evident based on developmental effects of fluoxetine on adult behavior of rodents, as well as studies of serotonin transporter (SERT) knockout mice and hypomorphic SERT alleles in humans (reviewed in [61]). This is consistent with rapidly emerging evidence that serotonergic innervation modulates the functional maturation of GABAergic neural circuits [62], [63], [64], [65], [66].

The critical period identified as important for immobility behavior in the FST (P29-35) matches a developmental period (P30-35) that is sensitive to social isolation stress in rats [59]. An earlier developmental origin of anxiety vs. emotion-related behavioral traits is corroborated by empirical evidence from human subjects, showing greatest risk for anxiety disorders in childhood, and a maximal risk for depressive disorders in adolescence [15], [16], [67], [68]. However, we caution that critical periods of developmental vulnerability are not necessarily identical with the ages at which these disorders first manifest themselves behaviorally or clinically.

Our analyses of corticogenesis failed to detect overt changes in the genesis of embryo-derived neurons in both γ2^+/−^ and γ2^−/−^ embryos, thereby likely excluding developmental processes that overtly affect the majority of neocortical neurons of γ2^+/−^ mice. However, these studies do not exclude deficits in numbers or activity-dependent differentiation of specific subtypes of GABAergic interneurons that have recently been implicated in MDD [69], [70]. Indeed, consistent with a role in controlling postnatal critical windows of vulnerability, GABAergic innervation is a protracted process that is regulated by network activity and known to extend well into the fifth postnatal week of mouse brain development [71], [72]. At developmental stages corresponding to the fourth and fifth postnatal week of the mouse, activity also controls the innervation field of cortical GABAergic interneurons [73]. A recent report suggests that neural activity of cortical GABAergic basket cells controls the density of initial perisomatic synaptic innervation of pyramidal cells, while GABA release in addition controls the subsequent pruning of GABAergic synapses [74]. It is conceivable therefore that GABAergic innervation is disturbed by the manipulations of GABAergic transmission used in our study. Moreover, GABA-potentiating anesthetics including BZs given to two-week-old mice have been reported to transiently increase dendritic spine formation of principal cells in the prefrontal cortex [75], [76]. These observations are consistent with electrophysiological evidence showing that GABA-mediated depolarization of immature neurons promotes the developmental functional maturation of glutamatergic synapses [77].

The identification of GABA_A_R function-dependent critical periods regulating anxiety- and emotion-related behavior in mice may help elucidate the developmental substrate of anxious depression in patients. Based on a large number of structural and functional parameters, the limbic and cortical brain areas of a ten day-old mouse (29 days post conception) correspond to those of a human fetus at the 143^rd^ and 197th day of gestation, respectively [48]. Based on the critical developmental period regulating ocular dominance plasticity, the visual cortex of a 23–33 day-old mouse corresponds to that of an approximately six-year-old human [33], [78]. Thus, based on the mouse data presented here the critical periods for anxiety- and depression-related behavior in humans is predicted to span the time from mid-gestation (anxiogenic effects of DZP) to school age (depressive-like effects of DZP). Future experiments will need to address whether DZP-regulated critical periods for anxiety- and emotional behavior can be mapped onto different neuroanatomical substrates, whether they are reflected in permanent changes in structure and function at the cellular level, and whether any such changes are reversible by antidepressant drug treatment.

## Supporting Information

Figure S1 Unaltered proliferation, migration and survival of γ2^−/−^ and γ2^+/−^ embryo-derived neocortical neurons. The density of neurons labeled with BrdU at different embryonic time points (E12.5 and 15.5) and accumulating in different embryonic and postnatal brain structures was determined using immunofluorescent staining of brain sections for BrdU or BrdU and NeuN and analyses by confocal microscopy. Timed pregnant females (γ2^+/−^ x γ2^+/−^ matings) were injected with BrdU at gestational day E12.5 (**a**) or E15.5 (**b–d**) and the brains harvested at E18.5 (a, b) or P21 (c, d). The density of BrdU-labeled neurons (cells/62500 µm^2^) that had migrated to the cortical subplate by E18.5 (a) and layer II/III (b) of γ2^+/−^ and γ2^−/−^ vs. γ2^+/+^ embryos was independent of genotype. Similarly, the density of E15.5 derived BrdU-positive cells or BrdU/NeuN double positive neurons that had accumulated in the neocortex (C) or hippocampal CA1, CA3 and dentate gyrus (DG) regions (d) of 3-week-old γ2^+/−^ vs. γ2^+/+^ mice was unaffected by genotype (c). Note that γ2^−/−^ mice exhibit a perinatal lethal phenotype that precluded their analyses in (c). Data indicate means ± SEM. n = 5/genotype, p>0.05 for all comparisons [Kruskal-Wallis (a, b) and Mann-Whitney (c, d)].
(DOCX)

Figure S2 Characterization of tamoxifen induced, CAGGCre-ER™-mediated recombination. Tamoxifen was injected into CAGGCre-ER™ X R26Y mice on P13 and P14 or P27 and P28 to induce recombination at the start of the third or fifth postnatal week, respectively and harvested at 6 weeks of age. **a**. Representative micrographs of sections through the dentate gyrus of CAGGCre-ER™ X R26Y mice treated with tamoxifen at the ages indicated. Scale bar, 50 µm. **b**. Quantitation of YFP positive cells in the dentate gyrus as a percentage of cells visualized by staining with the nuclear stain DRAQ5 (n = 4–6, p>0.05, Mann-Whitney).
(DOCX)

Figure S3 Analyses of TAM-induced recombination of the fγ2 locus in CAGGCre-ER™ X fγ2/+ mice by PCR of genomic forebrain DNA. Duplicate CAGGCre-ER™ X fγ2/+ mice were treated with TAM on P13/14 (lanes 1–4) or P27/28 (lanes 5, 6) and euthanized 24 (lanes1, 2) or 48 h later (lanes 3–6) as indicated. Untreated Emx1Cre X fγ2/fγ2 [1] and fγ2/+ mice (lacking a Cre transgene) were analyzed as positive and negative controls, respectively. The Emx1Cre transgene drives recombination of the fγ2 locus in the large majority of cells of the forebrain including glutamatergic neurons and glia but not GABAergic cells [1]. Genomic DNA (125 ng) from forebrain was subject to PCR using primers mapping to sites upstream and downstream of the 5′ and 3′ loxP site in the fγ2 locus, respectively, thereby allowing for simultaneous amplification of both the fγ2 locus and the recombined locus, fγ2Δ. Genomic DNAs of Emx1Cre X fγ2/fγ2 (lane 7) and CAGGCre-ER™ mice (lane 8) were amplified as positive and negative controls, respectively. The Cre loci of all samples were analyzed in parallel PCR reactions to verify the integrity of all DNA samples and as an internal standard. Note the bands of similar intensity representative of the fγ2Δ locus in DNA isolated 48 vs. 24 h after the second injection of TAM (lanes 1 and 2, vs. lanes 3 and 4) as well as 48 h after TAM injection o P27/28 (lanes 5,6). Abbreviations: γ2, region of *gabrg2* locus containing exon 8; fγ2, corresponding pseudo-WT locus containing lox P sites upstream and downstream of exon 8; fγ2Δ, *gabrg2* locus following Cre-mediated recombination and deletion of exon 8.
(DOCX)

Materials and Methods S1 GABAergic control of critical developmental periods for anxiety- and depression-related behavior in mice.
(DOCX)
