# Supplementary material for: GABAergic Control of Critical Developmental Periods for Anxiety- and Depression-Related Behavior in Mice
Source: PLoS One. 2012 Oct 11;7(10):e47441. doi: 10.1371/journal.pone.0047441 (PMC3469546; doi:10.1371/journal.pone.0047441)
Supplement: Figure S1 — Unaltered proliferation, migration and survival of γ2−/− and γ2+/− embryo-derived neocortical neurons. The density of neurons labeled with BrdU at different embryonic time points (E12.5 and 15.5) and accumulating in different embryonic and postnatal brain structures was determined using immunofluorescent staining of brain sections for BrdU or BrdU and NeuN and analyses by confocal microscopy. Timed pregnant females (γ2+/− x γ2+/− matings) were injected with BrdU at gestational day E12.5 (a) or E15.5 (b–d) and the brains harvested at E18.5 (a, b) or P21 (c, d). The density of BrdU-labeled neurons (cells/62500 µm2) that had migrated to the cortical subplate by E18.5 (a) and layer II/III (b) of γ2+/− and γ2−/− vs. γ2+/+ embryos was independent of genotype. Similarly, the density of E15.5 derived BrdU-positive cells or BrdU/NeuN double positive neurons that had accumulated in the neocortex (C) or hippocampal CA1, CA3 and dentate gyrus (DG) regions (d) of 3-week-old γ2+/− vs. γ2+/+ mice was unaffected by genotype (c). Note that γ2−/− mice exhibit a perinatal lethal phenotype that precluded their analyses in (c). Data indicate means ± SEM. n = 5/genotype, p>0.05 for all comparisons [Kruskal-Wallis (a, b) and Mann-Whitney (c, d)]. (DOCX) [file pone.0047441.s001.docx]

**Supporting Figure S1, Shen et al., GABAergic control of critical developmental periods for anxiety- and depression-related behavior in mice**

**
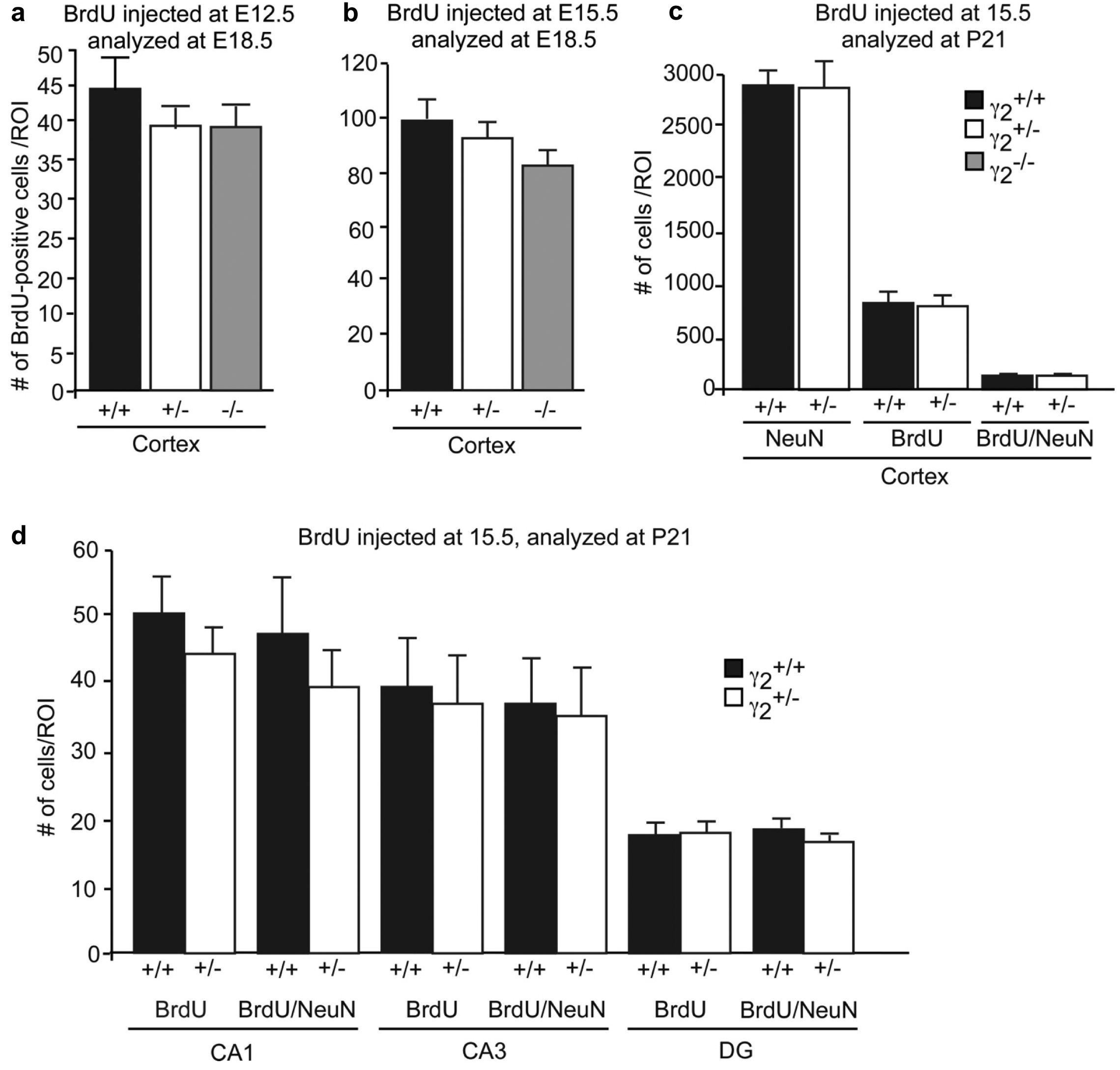
**

**Figure S1. Unaltered proliferation, migration and survival of** γ2^-/-^ **and** γ2^+/-^ **embryo-derived neocortical neurons.** The density of neurons labeled with BrdU at different embryonic time points (E12.5 and 15.5) and accumulating in different embryonic and postnatal brain structures was determined using immunofluorescent staining of brain sections for BrdU or BrdU and NeuN and analyses by confocal microscopy. Timed pregnant females (γ2^+/-^ x γ2^+/-^ matings) were injected with BrdU at gestational day E12.5 (**a**) or E15.5 (**b-d**) and the brains harvested at E18.5 (a, b) or P21 (c, d). The density of BrdU-labeled neurons (cells/62500 μm^2^) that had migrated to the cortical subplate by E18.5 (a) and layer II/III (b) of γ2^+/-^ and γ2^-/-^ vs. γ2^+/+^ embryos was independent of genotype. Similarly, the density of E15.5 derived BrdU-positive cells or BrdU/NeuN double positive neurons that had accumulated in the neocortex (C) or hippocampal CA1, CA3 and dentate gyrus (DG) regions (d) of 3-week-old γ2^+/-^ vs. γ2^+/+^ mice was unaffected by genotype (c). Note that γ2^-/-^ mice exhibit a perinatal lethal phenotype that precluded their analyses in (c). Data indicate means ± SEM. n = 5/genotype, p > 0.05 for all comparisons [Kruskal-Wallis (a, b) and Mann-Whitney (c, d)].
