# Supplementary material for: GABAergic Control of Critical Developmental Periods for Anxiety- and Depression-Related Behavior in Mice
Source: PLoS One. 2012 Oct 11;7(10):e47441. doi: 10.1371/journal.pone.0047441 (PMC3469546; doi:10.1371/journal.pone.0047441)
Supplement: Figure S2 — Characterization of tamoxifen induced, CAGGCre-ER™-mediated recombination. Tamoxifen was injected into CAGGCre-ER™ X R26Y mice on P13 and P14 or P27 and P28 to induce recombination at the start of the third or fifth postnatal week, respectively and harvested at 6 weeks of age. a. Representative micrographs of sections through the dentate gyrus of CAGGCre-ER™ X R26Y mice treated with tamoxifen at the ages indicated. Scale bar, 50 µm. b. Quantitation of YFP positive cells in the dentate gyrus as a percentage of cells visualized by staining with the nuclear stain DRAQ5 (n = 4–6, p>0.05, Mann-Whitney). (DOCX) [file pone.0047441.s002.docx]

**Supporting Figure S2, Shen et al., GABAergic control of critical developmental periods for anxiety- and depression-related behavior in mice**

**
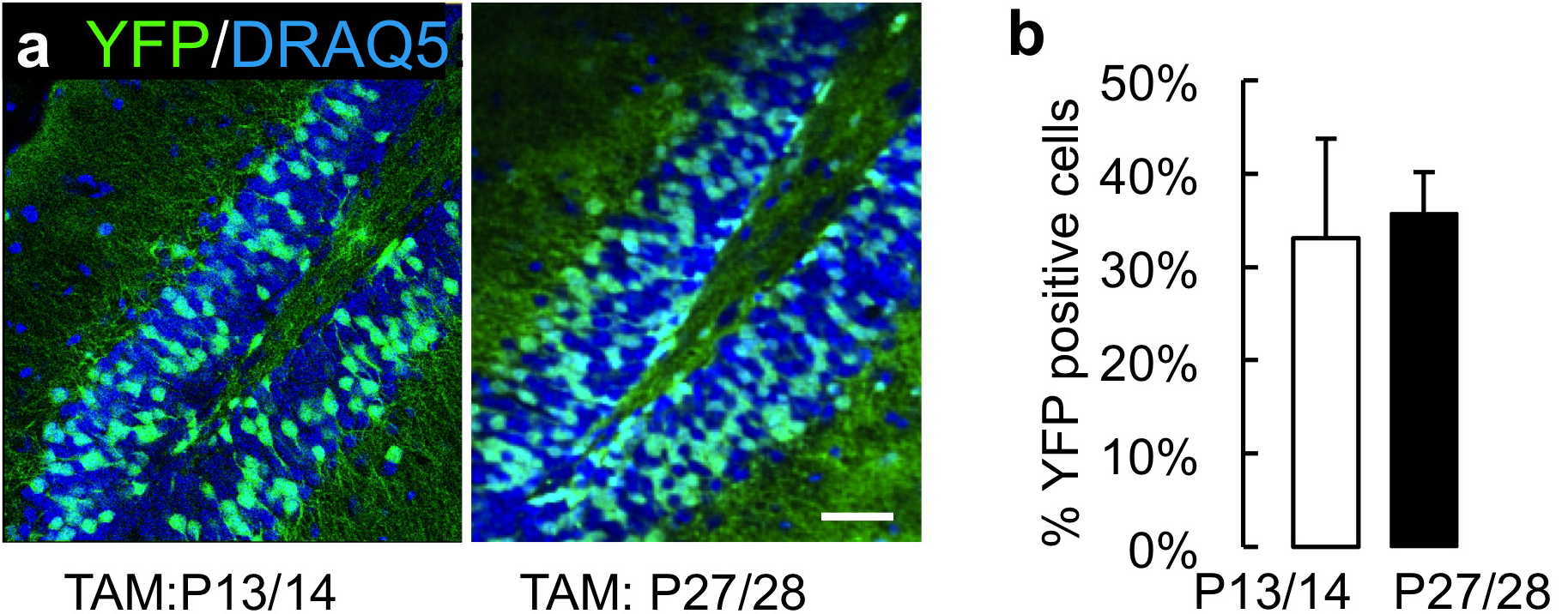
**

**Figure S2.** **Characterization of tamoxifen induced, CAGGCre-ER^TM^-mediated recombination**. Tamoxifen was injected into CAGGCre-ER^TM^ X R26Y mice on P13 and P14 or P27 and P28 to induce recombination at the start of the third or fifth postnatal week, respectively and harvested at 6 weeks of age. **a**. Representative micrographs of sections through the dentate gyrus of CAGGCre-ER^TM^ X R26Y mice treated with tamoxifen at the ages indicated. Scale bar, 50 μm. **b**. Quantitation of YFP positive cells in the dentate gyrus as a percentage of cells visualized by staining with the nuclear stain DRAQ5 (n = 4-6, p > 0.05, Mann-Whitney).
