# Supplementary material for: GABAergic Control of Critical Developmental Periods for Anxiety- and Depression-Related Behavior in Mice
Source: PLoS One. 2012 Oct 11;7(10):e47441. doi: 10.1371/journal.pone.0047441 (PMC3469546; doi:10.1371/journal.pone.0047441)
Supplement: Figure S3 — Analyses of TAM-induced recombination of the fγ2 locus in CAGGCre-ER™ X fγ2/+ mice by PCR of genomic forebrain DNA. Duplicate CAGGCre-ER™ X fγ2/+ mice were treated with TAM on P13/14 (lanes 1–4) or P27/28 (lanes 5, 6) and euthanized 24 (lanes1, 2) or 48 h later (lanes 3–6) as indicated. Untreated Emx1Cre X fγ2/fγ2 [1] and fγ2/+ mice (lacking a Cre transgene) were analyzed as positive and negative controls, respectively. The Emx1Cre transgene drives recombination of the fγ2 locus in the large majority of cells of the forebrain including glutamatergic neurons and glia but not GABAergic cells [1]. Genomic DNA (125 ng) from forebrain was subject to PCR using primers mapping to sites upstream and downstream of the 5′ and 3′ loxP site in the fγ2 locus, respectively, thereby allowing for simultaneous amplification of both the fγ2 locus and the recombined locus, fγ2Δ. Genomic DNAs of Emx1Cre X fγ2/fγ2 (lane 7) and CAGGCre-ER™ mice (lane 8) were amplified as positive and negative controls, respectively. The Cre loci of all samples were analyzed in parallel PCR reactions to verify the integrity of all DNA samples and as an internal standard. Note the bands of similar intensity representative of the fγ2Δ locus in DNA isolated 48 vs. 24 h after the second injection of TAM (lanes 1 and 2, vs. lanes 3 and 4) as well as 48 h after TAM injection o P27/28 (lanes 5,6). Abbreviations: γ2, region of gabrg2 locus containing exon 8; fγ2, corresponding pseudo-WT locus containing lox P sites upstream and downstream of exon 8; fγ2Δ, gabrg2 locus following Cre-mediated recombination and deletion of exon 8. (DOCX) [file pone.0047441.s003.docx]

**Supporting Figure S3, Shen et al., GABAergic control of critical developmental periods for anxiety- and depression-related behavior in mice**

**
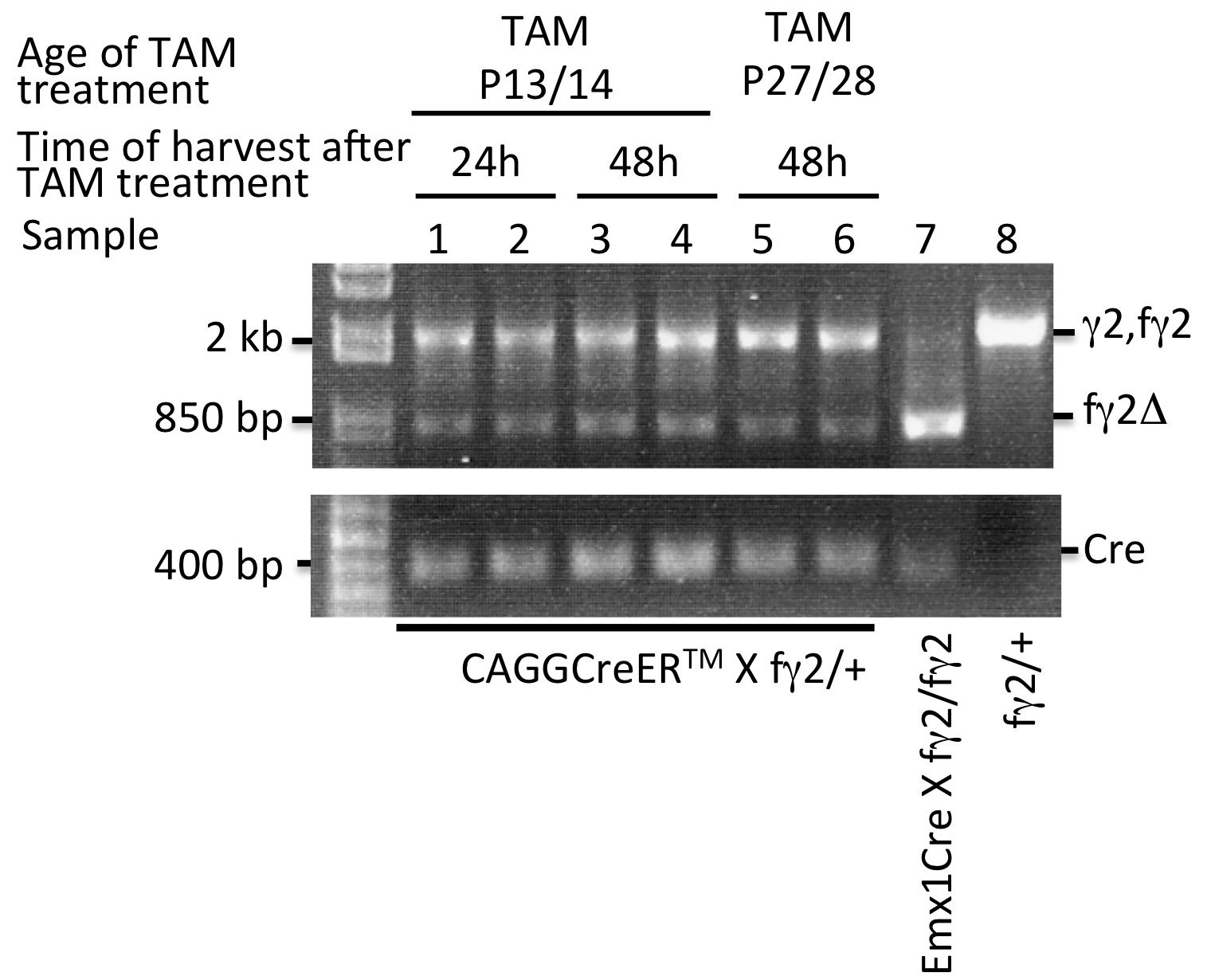
**

**Figure S3.** **Analyses of TAM-induced recombination of the fγ2 locus in CAGGCre-ER^TM^ X fγ2/+ mice by PCR of genomic forebrain DNA.** Duplicate CAGGCre-ER^TM^ X fγ2/+ mice were treated with TAM on P13/14 (lanes 1-4) or P27/28 (lanes 5, 6) and euthanized 24 (lanes1, 2) or 48 h later (lanes 3-6) as indicated. Untreated Emx1Cre X fγ2/fγ2 [[1](#_ENREF_1)] and fγ2/+ mice (lacking a Cre transgene) were analyzed as positive and negative controls, respectively. The Emx1Cre transgene drives recombination of the fγ2 locus in the large majority of cells of the forebrain including glutamatergic neurons and glia but not GABAergic cells [[1](#_ENREF_1)]. Genomic DNA (125 ng) from forebrain was subject to PCR using primers mapping to sites upstream and downstream of the 5’ and 3’ loxP site in the fγ2 locus, respectively, thereby allowing for simultaneous amplification of both the fγ2 locus and the recombined locus, fγ2Δ. Genomic DNAs of Emx1Cre X fγ2/fγ2 (lane 7) and CAGGCre-ER^TM^ mice (lane 8) were amplified as positive and negative controls, respectively. The Cre loci of all samples were analyzed in parallel PCR reactions to verify the integrity of all DNA samples and as an internal standard. Note the bands of similar intensity representative of the fγ2Δ locus in DNA isolated 48 vs. 24 h after the second injection of TAM (lanes 1 and 2, vs. lanes 3 and 4) as well as 48h after TAM injection o P27/28 (lanes 5,6). Abbreviations: γ2, region of *gabrg2* locus containing exon 8; fγ2, corresponding pseudo-WT locus containing lox P sites upstream and downstream of exon 8; fγ2Δ, *gabrg2* locus following Cre-mediated recombination and deletion of exon 8.

1. Earnheart JC, Schweizer C, Crestani F, Iwasato T, Itohara S, et al. (2007) GABAergic control of adult hippocampal neurogenesis in relation to behavior indicative of trait anxiety and depression states. J Neurosci 27: 3845-3854.
