# Supplementary material for: GABAergic Control of Critical Developmental Periods for Anxiety- and Depression-Related Behavior in Mice
Source: PLoS One. 2012 Oct 11;7(10):e47441. doi: 10.1371/journal.pone.0047441 (PMC3469546; doi:10.1371/journal.pone.0047441)
Supplement: Materials and Methods S1 — GABAergic control of critical developmental periods for anxiety- and depression-related behavior in mice. (DOCX) [file pone.0047441.s004.docx]

**Supporting Materials and Methods, Shen et al., GABAergic control of critical developmental periods for anxiety- and depression-related behavior in mice**

**Analyses of TAM-induced recombination of the f**γ**2 locus using PCR of genomic DNA**

Genomic DNA was isolated and purified from forebrain of all mice using proteinase K digestion followed by phenol chloroform extraction. DNA aliquots (125 ng) were subject to PCR (31 cycles) using the forward primer fγ2upper2 (mapping to an intronic sequence upstream of the 5’ loxP site in the fγ2 locus [[1](#_ENREF_1)], 5’-GCCTG ATTGT GGAAA TAAAA-3’) and the reverse primer γ2i89-1 (mapping to a intronic sequence downstream of the 3’ lox P site of the fγ2 locus, 5’-ATGCC TGCAT AAACA AACAC-3’). The Cre loci (CAGGCre-ER^TM^ and Emx1Cre, respectively) were amplified as a reference in parallel PCR reactions containing the same DNA aliquots and using primers NesCreF (5’ATTTG CCTGC ATTAC CGGTC-3’) and NesCreR (5’-ATCAA CGTTT TCTTT TCGG-3’). PCR reactions were analyzed by standard agarose gel electrophoresis.

**Analyses of generation, migration and survival of embryo-derived neurons**. Timed pregnant female mice of γ2^+/-^ x γ2^+/-^ or WT x γ2^+/-^ matings were injected with BrdU (50 mg/kg/day, Sigma; St Louis, MO) at gestational day (E)12.5 or E15.5, respectively. The embryos (both sexes) were harvested at E18.5 and the brains post-fixed in 4% paraformaldehyde in 0.1M phosphate buffer (pH 7.4) for 48 h. A second group of pregnant females were injected analogously at gestational day 15.5 and the offspring anesthetized with Avertin and perfused at postnatal (P) day 21 with 4% paraformaldehyde in 0.1 M phosphate buffer (pH 7.4), post-fixed in the same solution (18 h, 4ºC) and stored in PBS at 4ºC until use. The brains were sectioned coronally (50 μm) using a Vibratome (Vibratome, St. Louis). Free-floating sections were permeabilized with 1% Triton X-100 in PBS, incubated in 2N HCl for 30 min at 37ºC and washed 5 min in 0.1M sodium borate (Na_2_B_4_O_7_) at room temperature followed by three to five washes of 5 min in PBS. They were stained with rat polyclonal antibody against BrdU (1:500; Accurate Chemical, Westbury, NY) and mAb anti-NeuN (1:1000; Chemicon, Temecula, CA) in blocking solution containing normal donkey serum. Sections were developed with Cy3- and Alexa 488-conjugated secondary antibodies (Jackson ImmunoResearch, West Grove, PA) mounted onto glass coverslips and imaged by confocal microscopy. Z-plane sectioning (1 µm steps) was performed to ensure colocalized signals belonged to the same cells. BrdU and NeuN single- and double-positive neurons in the regions of interest (250 X 250 μm) were quantified in regions of interest (250 X 250 μm) of three sections per brain and five brains for each condition, using Image J software.

1. Schweizer C, Balsiger S, Bluethmann H, Mansuy M, Fritschy JM, et al. (2003) The gamma2 subunit of GABA(A) receptors is required for maintenance of receptors at mature synapses. Mol Cell Neurosci 24: 442-450.
